# Synthesis, physicochemical properties and ocular pharmacokinetics of thermosensitive *in situ* hydrogels for ganciclovir in cytomegalovirus retinitis treatment

**DOI:** 10.1080/10717544.2017.1413448

**Published:** 2017-12-12

**Authors:** Qiyue Wang, Chunmeng Sun, Bohui Xu, Jiasheng Tu, Yan Shen

**Affiliations:** ^a^ Center for Research Development and Evaluation of Pharmaceutical Excipients and Generic Drugs, Department of Pharmaceutics, China Pharmaceutical University Nanjing China; ^b^ School of Pharmacy, Nantong University Nantong China

**Keywords:** *In situ* hydrogels, PBLA-PEG-PBLA, thermosensitive, ganciclovir, intravitreal injection

## Abstract

Ganciclovir (GCV) is one of the most widely used antiviral drugs for the treatment of cytomegalovirus (CMV) retinitis. In this context, the aim of this study was to design *in situ* thermosensitive hydrogels for GCV ocular delivery by intravitreal injection to achieve sustained drug release behavior and improved ocular bioavailability in the treatment of CMV retinitis. A thermosensitive poly-(β-butyrolactone-co-lactic acid)-polyethylene glycol-poly (β-butyrolactone-co-lactic acid) (PBLA-PEG-PBLA) triblock copolymer was synthesized by ring-opening polymerization and characterization. The GCV-loaded PBLA-PEG-PBLA *in situ* hydrogels (15%, *w/w*) were then prepared with drug concentration at 2 mg·mL^−1^ and the gelation temperatures, rheological properties, *in vitro* degradation and syringeability of *in situ* hydrogels for intravitreal injection were also investigated. Membraneless dissolution model was used to explore drug release behavior of PBLA-PEG-PBLA *in situ* hydrogel. The results indicated that more than 45 and 85% of GCV can be released within 24 and 96 h, respectively, which was verified by a non-Fickian diffusion mechanism. *In vivo* ocular pharmacokinetics study showed that area under drug-time curve (AUC) and half-life of PBLA-PEG-PBLA *in situ* hydrogel was higher (AUC was 61.80 μg·mL^−1^·h (*p* < .01) and *t*_1/2_ was 10.29 h in aqueous humor; AUC was 1008.66 μg·mL^−1^·h (*p* < .01) and *t*_1/2_ was 13.26 h (*p* < .01) in vitreous) than GCV injection with extended therapeutic activity. Based on obtained results, it was concluded that the thermosenstive PBLA-PEG-PBLA *in situ* hydrogel is a promising carrier of GCV for intravitreal injection.

## Introduction

Human cytomegalovirus (HCMV) is a kind of DNA virus that belongs to the *β*-subgroup of herpes viruses. HCMV infection spreads widely in high-density population and usually shows recessive infection. Mostly infected patients do not show any symptoms. However, under certain conditions, cytomegalovirus (CMV) could infect multiple organs and become life-threatening (Freeman et al., 1984; Hinkle et al., 2000). CMV retinitis is a serious ophthalmic disease that may cause blindness. CMV could travel in retinal blood vessels and spread outward through the compact capillary wall of retinal to infect the retinal pigmented epithelium. The resulted disorganized retinal pigmented epithelium may break down outer blood–retina barrier and leads to retinal hemorrhage, detachment and blindness. (Nussenblatt & Lane, 1998; Janoria et al., 2009; Martin et al., 2002).

Ganciclovir (GCV) is a new type of selective nucleoside antiviral drugs, which exhibits potent antiviral activity against herpes simplex virus (HSV) and CMV (Cantrill et al., 1989; Choopong et al., 2010). GCV inhibit viral replication mainly through two mechanisms: (1) GCV triphosphate inhibits viral DNA polymerase, the formation of deoxyguanosine triphosphate, and hence the replication of virus. (2) GCV triphosphate is directly combined with DNA chains and prevents further viral replication. Conventional ophthalmic solutions of GCV are available but limited in the treatment of CMV retinitis due to low bioavailability (Chien, 1991; Mitra, 1993). Similarly, oral delivery and intravenous injection are limited in efficacy due to low drug concentration at infection site and off-target adverse effect such as a bone-marrow suppressive effect. (Adibkia et al., 2007). Therefore, local intravitreal therapy has been proposed as a safe and well-tolerated alternative route for the treatment of retinitis infections. However, due to the short half-life of the drug, frequent intravitreal injections are necessary to maintain therapeutic levels of GCV. These continuous administrations have the risk of emerging in cataracts, retinal detachments, hemorrhages or endophthalmitis (Cochereau-Massin et al., 1991).

In order to overcome these drawbacks, some ophthalmic implants have been developed as a topical treatment option (Choonara et al., 2006). However, these implants are not usually biodegradable and require surgical implantation and retirement. Furthermore, those systems may produce long-term side effects such as retinal detachment, endophthalmitis and vitreous hemorrhage (Bourges et al., 2006).

Another possibility to minimize these problems can consist on the administration of GCV in biodegradable carriers. In this study, GCV-loaded *in situ* biodegraded hydrogels have been proposed. On the one hand, it appears that *in situ* hydrogels can provide effective intravitreal concentrations of different kind of drugs with no signs of ocular toxicity just like mitomycin-loaded poly(trimethylenecarbonate)-Pluronic F127-poly(trimethylenecarbonate) (PTMC-Pluronic F127-PTMC) *in situ* hydrogels can sustained release for 15 days, whereas the injection of a similar drug solution shows shorter eye concentration above the IC_50_ (Xi et al., 2014). On the other hand, drug-loaded biodegradable *in situ* hydrogels did not show any retinal or adjacent tissues toxicity, following either electroretinography or histological studies (He et al., 2008; Smith et al., 1992).

Hydrogel is a kind of hydrophilic system which can absorb a large number of moisture while in a semi-solid form (Hoffman, 2002; Huynh et al., 2011). Environment-sensitive hydrogels can show characteristic response even after small changes in physical (temperature, electromagnetic field, etc.) or chemical conditions (pH, ionic strength, enzyme, etc.) (Tokarev & Minko, 2010; Doring et al., 2013; Wen et al., 2013; 2014a, 2014b; Xu et al., 2014a). The thermosensitive hydrogel was a liquid form at room temperature which was easy to inject and shows a gelatinization as the temperature increasing. Solidified hydrogels could load drugs in their net structures and demonstrate a sustained release behavior which can reduce the injection frequency.

The aim of this study was to design and develop thermosensitive *in situ* hydrogel-based delivery systems for the intravitreal administration of GCV. For this purpose, the hydrogel was prepared by poly (β-butyrolactone-co-lactic acid)-polyethylene glycol-poly (β-butyrolactone-co-lactic acid) (PBLA-PEG-PBLA) triblock copolymer, which was synthesized by ring-opening polymerization. In addition, the gelation temperature, gelation time, rheological properties, syringeability, *in vitro* drug release, and *in vivo* ocular pharmacokinetics of these systems as controlled release dosage forms was also evaluated.

## Materials and methods

### Materials

d,l-Lactide (LA, 99.5%) was purchased from Jinan Daigang Biotechnology Co. Ltd (Jinan, China). *β*-Butyrolactone (β-BL, ≥98%), PEG_1500_ and stannous 2-ethylhexanoate (stannous octoate, 95%) were purchased from Sigma (Shanghai, China). GCV (GCV, ≥98%) was purchased from Nanjing Duly Biotechnology Co. Ltd (Nanjing, China).

### Animals

Adult male New Zealand white rabbits weighing between 2.0 and 2.5 kg were obtained from the Experimental Animal Center of Nanjing Qinglongshan. All animal experiments were conducted in accordance with the Guide for Laboratory Animal Facilities and Care and under approval by Animal Ethics Committee of China Pharmaceutical University.

### Synthesis and characterization of PBLA-PEG-PBLA copolymer

#### Synthesis of the PBLA-PEG-PBLA copolymer

The PBLA-PEG-PBLA copolymer was synthesized by ring-opening polymerization of DL-LA and *β*-BL in the presence of PEG (Xu et al., 2014b; Xu et al., 2015). Briefly, the PEG_1500_ (4.0 g) and d,l-LA (9.0 g) were added into a dried three-necked flask equipped with reflux condensing tube, and heated to 130 °C in vacuum with stirring for 2 h to remove water before addition of *β*-BL (1.0 g) and Sn(Oct)_2_ (40 mg, 32 μL). The reaction was carried out at 150 °C for 8 h under N_2_ atmosphere. The obtained product was washed with distilled water at 70 °C several times to remove unreacted monomer, freeze dried and then stored at 4 °C.

#### ^1 ^H NMR analysis

^1 ^H NMR was used to demonstrate the structure of PBLA-PEG-PBLA copolymer and to calculate the block ratio and *M_n_* of copolymer. ^1 ^H NMR spectra were recorded at room temperature with AV-300 NMR spectrometer (Bruker Corp., Switzerland). The PBLA-PEG-PBLA copolymer was dissolved in CDCl_3_. Chemical shifts were given in ppm using tetramethylsilane as an internal reference.

#### Gel permeation chromatography (GPC) analysis

GPC (RID-10 A detector, LC-20AT pump, LC solution workstation, LC solution GPC re-analysis software, Shimadzu Co. Let., Japan) was used to determine the *M_w_* and *M_w_* distribution of PBLA-PEG-PBLA copolymers. Tetrahydrofuran was used as eluent with a flow rate of 1.0 mL·min^−1^ at 40 °C. The measurements of copolymers were calibrated with monodisperse polystyrene standards (ZZBIO co., Ltd, Shanghai, China).

### Investigation of PBLA-PEG-PBLA copolymer gel solutions property

#### Rheological properties

Rheological properties of PBLA-PEG-PBLA copolymer solutions were determined according to reported method (Grassi et al., 2006; Andrews et al., 2009). In this study, a Discovery HR-3 rheometer (TA Instruments, New Castle, DE) equipped with a 40 mm diameter parallel geometry was used. An appropriate strain of oscillation analysis was determined through assessing the linear viscoelastic region. PBLA-PEG-PBLA copolymer solutions (*M_w_* 6244, PEG:PBLA = 1:2.5, DL: BL = 9:1) (10, 15, and 20%, *w/w*) were applied to the lower plate with a predefined distance in between two plates of 500 μm. The samples were allowed to equilibrate for 5 min at 25 °C and then heated from 25 °C to 40 °C at a rate of 1 °C·min^−1^ and a frequency of 1 Hz. Storage modulus, loss modulus and viscosity were recorded under different temperature. GCV PBS solutions (pH7.4, GCV concentration 2 mg·mL^−1^) were tested as control.

#### Syringeability

The syringeability (Cilurzo et al., 2011) was assessed as the force required to inject copolymer solutions (10, 15, and 20%, *w/w*) at 25 °C. The measurement of the injection force was performed in a compression mode by using a software-controlled TA.XT-Plus texture analyzer (Stable Micro System Ltd., Britain). The 1 mL size syringes with different needles were positioned in the dynamometer holder, keeping needle downward. The plunger end of the syringe was placed in contact with a cylindrical analytical probe (Type P/50 R). To mimic the manual syringe delivery to patient, the test was conducted at the crosshead speed of 1 mm/s at the distance of 20 mm. The loading force required to displace the plunger was measured (N) as a function of plunger displacement (mm) at the induction force of 0.002 N. GCV solutions were tested as control. All the experiments were conducted in triplicates.

### In vitro degradation of PBLA-PEG-PBLA in situ hydrogels

One milliliter of the PBLA-PEG-PBLA copolymer solutions (10, 15, and 20%, *w/w*) in test tubes (15 mm × 150 mm) were incubated in a thermostatic water bath at 37 °C for 10 min till complete gel formation. Then, 4 mL of PBS (pH 7.4) was added to each tube and shaken at 100 rpm by the SHZ-82 A thermostatic water bath oscillator (GuoHua Electrical Appliance Co., Ltd., China) during the entire period of degradability studies. The PBS from each tube were collected into a beaker and replaced with same volume of fresh PBS every 2 days. At each time intervals (0, 1, 4, 7, 14, 21, and 28 d), the remaining hydrogels and the withdrawn buffer solutions were lyophilized by FD-2 A freeze dryer (BIOCOOL Co. Ltd., China), weighed and then analyzed by GPC for *M_w_*. The amount of remaining copolymer (*W_r_*) was calculated as Equation (1):

(1) $$W_{r}=W_{t}-W_{s}-W_{n}$$

where *W_t_* is the total weight of test tube with lyophilized hydrogels, *W_n_* is the weight of the test tube and *W_s_* is the salt contents in the remaining gels which were calculated by the difference in weights between the salts added in the tube and the sum of salts and dissolution gel remained in the replaced buffer solutions during the period of experiment.

### Preparation and characterization of GCV thermosensitive in situ hydrogels

GCV thermosensitive *in situ* hydrogels were prepared according to the following process. The formulation of *in suit* hydrogel is shown in Table S1. PBLA-PEG-PBLA copolymer (1.5 g) and GCV (20 mg) were dissolved in 10 mL of phosphate buffer (pH = 8.0, 0.05 M) with stirring. Mannitol and sodium chloride were added to adjust osmotic pressure followed by passing through a microporous membrane filter (0.22 μm).

The pH and osmotic pressure of GCV *in situ* hydrogels were measured by the PHS-3 C pH meter (Lei Ci Instrument, China) and STY-1 osmometer (Tian Da Tian Fa Instrument, China). Gelation, rheological, and syringeable properties of GCV *in situ* hydrogels were determined as per aforementioned method.

### In vitro release

Membraneless dissolution model was used to determine *in vitro* drug release behavior of *in situ* hydrogel of GCV. Two milliliters of hydrogel solution was taken into a test tube (15 mm × 150 mm) and incubated in thermostatic water bath at 37 °C for 10 min till gel was formed completely. After that, 4 mL of phosphate buffer solution (pH 7.4) containing 1% *w/w* of hyaluronic acid was added to the test tube as a release medium and the hyaluronic acid was used to simulate the high viscosity of vitreous. The tube was shaken at 100 rpm during the entire release period. The samples were withdrawn at predefined intervals of time and replaced with equal volume of fresh release medium. The collected samples were filtered before HPLC analysis to calculate cumulative drug release. The HPLC condition was described as follows: mobile phase was 0.02 M CH_3_COONH_4_–methanol (95:5, *v/v*, pH 4.5); injection volume was 20 µL; column temperature was 35 °C; flow speed was 1.0 mL·min^−1^; detection wavelength was 254 nm.

### Pharmacokinetics of GCV-loaded thermosensitive gel

Forty-two rabbits were divided into test group and reference group equally. Male, New Zealand white rabbits were anesthetized by an *i.p.* injection of 20% (*w/w*) urethane solution (5 mL·kg^−1^). Eyeballs of each rabbit were anesthetized locally with procaine eye drops. Then, 0.1 mL of *in suit* hydrogel solution (15%, *w/w*) and reference solution were injected into the vitreous cavity of test group and reference group separately with injection site 4 mm away from corneal limbus. The rabbits were euthanized at 2, 6, 12, 24, 48, 96, and 144 h after injection. Aqueous and vitreous were collected and pretreated with equal volume of methanol to precipitate proteins. The supernatant were collected by centrifugation and subjected to HPLC analysis. The HPLC condition was describe as follow: mobile phase was 0.01 M CH_3_COONH_4_–acetonitrile (99.8:0.2, *v/v*); injection volume was 20 µL; column temperature was 30 °C; flow speed was 1.0 mL·min^−1^; detection wavelength was 254 nm.

### Statistical analysis

All *in vitro* experiments were carried out in triplicates. The results were presented as means ± SD. Statistical comparisons were evaluated using the Student’s *t*-test or one-factor analysis of variance. *p* < .05 was accepted as significance.

## Results and discussion

### ^1^H NMR and GPC spectra of PBLA-PEG-PBLA copolymer

**Figure 1.**
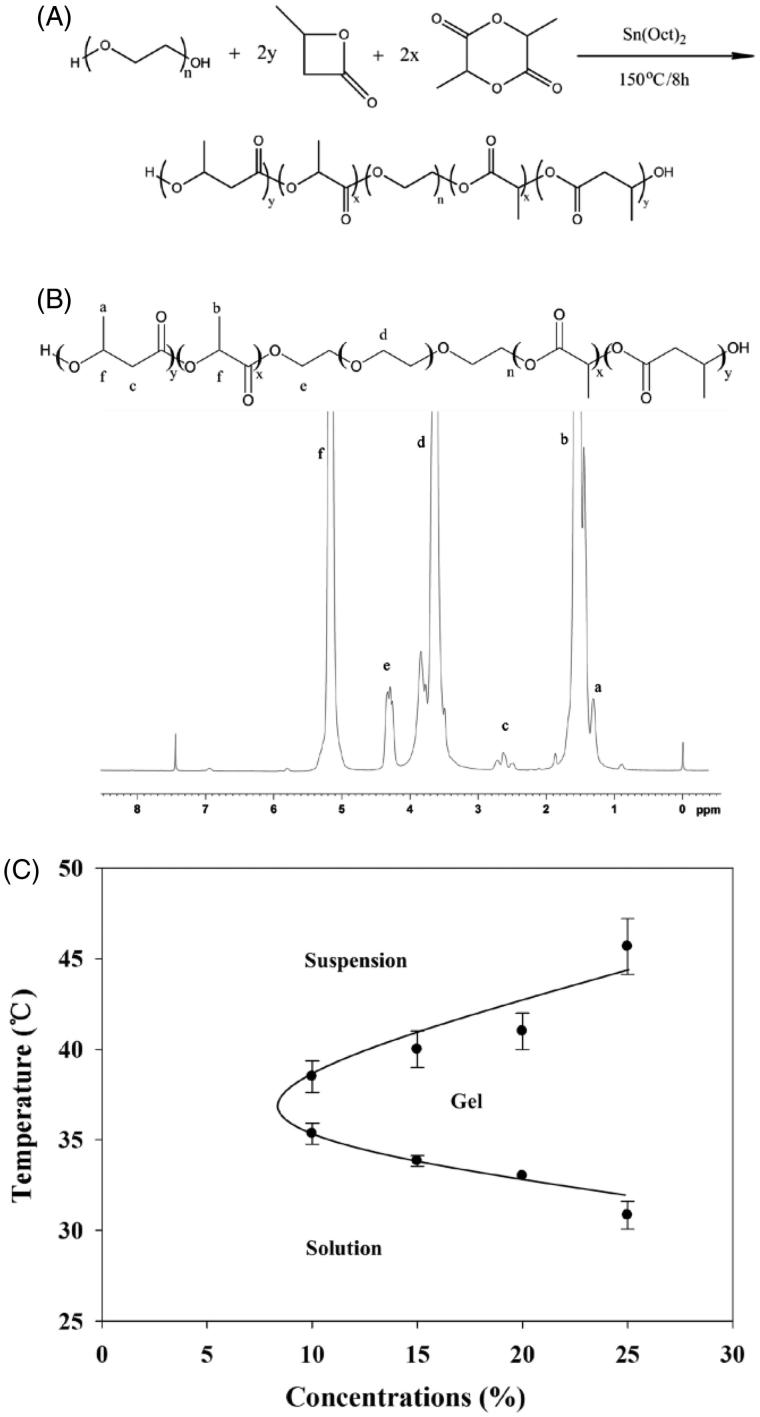
(A) Synthesis of PBLA-PEG-PBLA triblock copolymers. (B) ^1^H NMR spectrogram of PBLA-PEG-PBLA. (C) Phase diagrams of the PBLA-PEG-PBLA copolymer aqueous solutions.

The synthesis steps and ^1 ^H NMR spectra of PBLA-PEG-PBLA triblock copolymers is shown in Figure 1(a,b), respectively. The peak at 1.56 and 1.31 ppm shown the DL-LA and *β*-BL was participated in the ring-opening polymerization process. The *M_n_* and *M_w_* of PBLA-PEG-PBLA were calculated by ^1 ^H NMR spectra and GPC, respectively. The *M_n_* was 4263 g·mol^−1^ and *M_w_* was 6244 g·mol^−1^ with a polydispersity index of 1.464 (Table S2), suggesting a narrow distribution of synthesized PBLA-PEG-PBLA copolymer was achieved. The difference of *M_w_* and *M_n_* may be caused by unreacted monomer including d,l-LA, *β*-BL and oligomer as well as degradation products. Degradation products may be produced due to the temperature increase by washing copolymers in hot water, which makes the *M_n_* lower than *M_w_*.

### Gelation temperature and gelation time

The gelation temperatures of copolymer solutions were determined by vial inverting method (see Supporting information). The PBLA-PEG-PBLA copolymer solution exhibited a sol–gel phase transition and gel-precipitation phase transition as the temperature increasing (Figure 1(c)). Previous researches indicated that the sol–gel phase transition mechanism of copolymer solution is Micellar bridging effect between the micelles (Schindler et al., 1982). The PBLA-PEG-PBLA copolymers formed micelles in water with a hydrophobic core of PBLA and a hydrophilic shell of PEG. The hydrogen bonds between water molecules and PEG blocks were the main force to maintain the solubility of micelles. As the temperature increased, the PEG blocks slowly dehydrated and became less soluble because the hydrogen bonds were destroyed, eventually leading to aggregation of micelles and transition from solution to hydrogel. Gelation time was also investigated by the different method (Mathew et al., 2014) (see Supporting information). For the reversing method, 10% (*w/w*) copolymer solution can from hydrogel at 30.17 ± 4.58 s, compared to 6.67 ± 1.51 s for 20% (*w/w*) copolymer solution. The gelation time was reduced as the concentration of PBLA-PEG-PBLA copolymer was increasing (Table S3), which indicated that higher polymer concentration could form hydrogel faster leading to stronger anti-dilution ability. With stronger anti-dilution ability, drug could be easily locked in the hydrogel matrix, which significantly reduce the burst release and prolonged the retention time in the injection site.

The gelation temperature decreased as the concentrations of copolymer increased in the tested concentration range of 10 to 25% (*w/w*). PBLA-PEG-PBLA copolymer solution at concentration higher than 25% *(w/w)* easily formed hydrogel at room temperature and could not be injected due to its high viscosity. The critical gelation concentration (CGC) was defined as lowest concentration that copolymer solution can form hydrogel with temperature increasing and the range of CGC of PBLA-PEG-PBLA is from 5 to 10% (*w/w*). Not only the CGC, but also the gelation temperature effect by the molecular weight and ratio of copolymer. Phase behaviors of the copolymer solution, including sol–gel transition temperature, hydrogel window width and CGC are all dependent on molecular structure and *M_w_* of triblock copolymer, and can be adjustable (Xu et al., 2014b). With the molecular weight increased, gelation temperature shows significant increase and gelation window width was narrower. The PEG/PBLA ratio should be in a narrow range which between 1:2 and 1:2.5 could be the appropriate gelation temperature and suitable CGC.

### Rheology, syringeability and morphological observation

**Figure 2.**
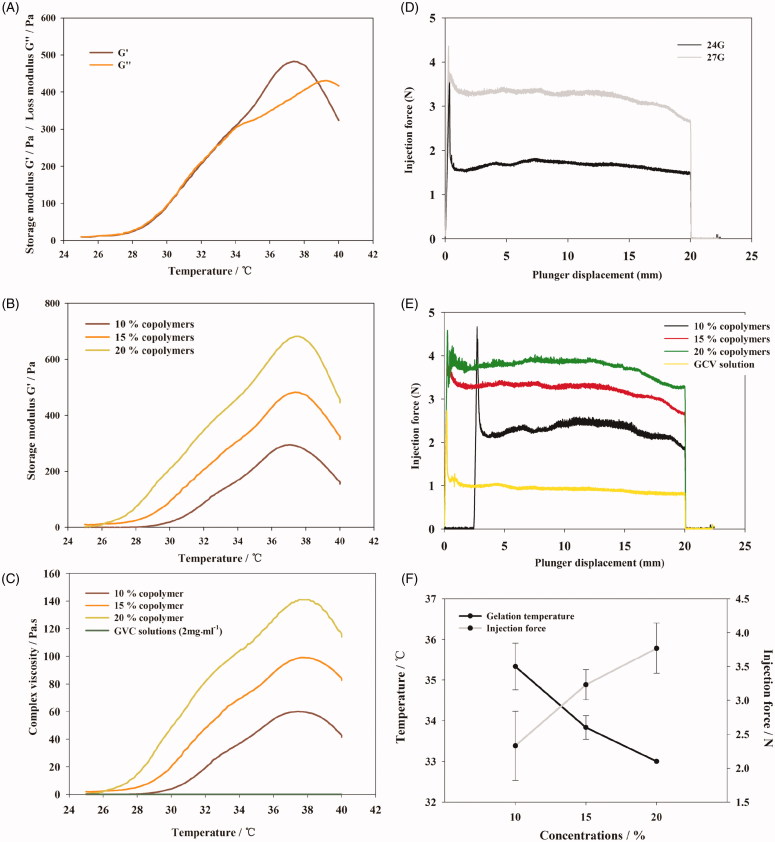
(A) Phase transition process of copolymer solutions (15%, *w/w*) characterized by the variance of storage modulus and loss modulus as a function of temperature. (B) Storage modulus of copolymer solutions at different concentration as a function of temperature. (C) Viscosity of copolymer solutions at different concentration as a function of temperature. (D) Required force to inject copolymer solution (15%, *w/w*) from 1 mL syringes equipped with 24 G × 20 mm, 27 G× 12.9 mm needles into air at the crosshead speed of 1 mm·s^−1^. (E) Required force to inject copolymer solutions at different concentration from 1 mL syringes equipped with 27 G × 12.9 mm needles into air at the crosshead speed of 1 mm·s^−1^. (F) Variation trend of gelation temperature and required force to inject copolymer solutions on different concentration of PBLA-PEG-PBLA solution.

Rheological study was conducted to explore microscopic or internal structural change of thermosensitive gels under gradual heating process. The linear viscoelastic region was analyzed and a strain of 0.1% was determined before oscillatory tests. The storage modulus (G′) and loss modulus (G″) are two important parameters to describe viscoelasticity. G′ is a measure of the elasticity while G″ represents viscous components. Oscillation temperature sweep of copolymer solution (15%, *w/w*) was displayed in Figure 2(a). The G′ and G″ were both low when temperature below 30 °C and G′ was lower than G″ which represent the system were still maintain the liquid form. G″ was little increased with temperature increasing because numbers of micelles formed by the copolymer were increased. When the temperature was higher than 30 °C, both G′ and G″ increased significantly with the increasing temperature. G′ increased faster than G″ because the polymer system become sufficiently concentrated so that micelle begins to be caged by its neighbors (Grassi, et al., 2006) and form the gel structure. The two curves intersected at a temperature which was the sol–gel phase transition temperature (Ricci et al., 2002; Andrews et al., 2009) of the copolymer solution. The sol–gel phase transition temperature detected by rheological method was close to the value determined by the vial inverting method (Table S3). Beyond the sol–gel transition temperature, G′ and G″ reached a plateau and then decreased because the hydrogel structure was destroyed with the increase in temperature. Two curves intersected at a higher temperature which was the gel-precipitation phase transition temperature of the copolymer solution and the hydrogel was then transformed into a flowing state.

To optimize the concentration of copolymer solution to resist shearing motion caused by eyeball movement, the gel strength of copolymer solutions of 10, 15, and 20% (*w/w*) was compared (Figure 2(b)). As an indicator of gel strength, G′ significantly increased as the copolymer concentration increased, suggesting hydrogel of higher copolymer solution can prolong the ocular retention.

However, hydrogel of higher concentration demonstrated higher viscosity and lower syringeability (Figure 2(c)). The viscosity of PBLA-PEG-PBLA copolymer solutions from 10 to 20% was slightly higher than GCV aqueous injection at room temperature, while increased rapidly as the temperature increasing and also showed an obviously demonstrated a concentration-dependent manner. The viscosity of 20% PBLA-PEG-PBLA copolymer solution was reached 140 Pa·s with a higher increasing rate while 10% copolymer solution gave a highest viscosity of 60 Pa·s with a slower increasing rate. The results show that lower concentration has the better injectable property but less strong hydrogel strength, which mean the final concentration of copolymer solution need to balance the effects of different factors.

The syringeability was evaluated as the injection force since it is an important parameter for injectable *in situ* hydrogels. The injection force is divided into three parts: (a) overcoming the resistance force of the syringe plunger; (b) imparting kinetic energy to the liquid; and (c) forcing the liquid through the needle (Allahham et al., 2004). The main parameters of syringeability studies are plunger-stopper breakloose force (PBF), maximum force and dynamic glide force (DGF) (Voigt et al., 2012). PBF refers to the force required to initiate the movement of the plunger; maximum force (*F*_max_): the highest force measured during the course of injection which was always equal to the PBF; DGF was referred to the force required to sustain the movement of the plunger.

Injection force was strongly affected by injection through different sizes of needles. From Figure 2(d), the PBF and DGF of injection using 27-G needle were significantly higher compared to 24-G needle. The value of *F*_max_ and PBF were equivalent during the whole injection course, which indicated the resistance force was highest at the beginning of injection. American Academy of Ophthalmology suggests to use needles of 27-G or smaller with a length between 1/2 to 5/8 inch to minimize patients’ discomfort and eye damage (Aiello et al., 2004; Avery et al., 2014).

DGF shows a concentration-dependent manner while the change of PBF was not regular. As shown in Figure 2(e), higher PBLA-PEG-PBLA copolymer concentration would significantly increase the DGF. On the other hand, the DGF of GCV aqueous injection was smaller than the copolymer solution (*p* < .01), which indicated that the decrease in the concentration of copolymer is beneficial for injection.

In order to ensure the syringeability will meet the requirement which the upper limit of injection force was set at 10 N and which is the lower the better in treatment process (Cilurzo et al., 2011; Voigt et al., 2012), the concentration of copolymer solution should be as low as possible. After contract the variation trend of gelation temperature and required force of copolymer solutions on different concentration (Figure 2(f)), the result shows that the variation trend was reversed with concentration increase.

To observe the hydrogel formation during injecting process (see Supporting information), PBLA-PEG-PBLA copolymer solutions were injected into PBS solutions at 37 °C. As shown in Figure S1A, the copolymer solution with concentration of 15 and 20% can directly form semi-solid hydrogel after injecting which indicated the rapid gelatinization process. But, with concentration lower than 15%, copolymer solution was easily diluted and hard to form hydrogel efficiently. Morphology of PBLA-PEG-PBLA *in situ* hydrogels was then observed through light microscope (Figure S1B). After the hydrogel was formed from the copolymer solution (15%, *w/w*) and dried by BHX electrothermal oven (Keer equipment Pty. Ltd, China), the observably pore structure were shown which caused by the moisture evaporation. Less porous and irregular holes on the surface were observed for higher copolymer concentration (20%, *w/w*), result in retarding the burst release of drugs. It was speculated that more amount of copolymer incorporated to form higher copolymer concentration could block the pore to achieve the sustained release behavior compared to the lower copolymer concentration (10%, *w/w*).

Considering the gelation temperature of copolymer solutions with concentrations at 15 and 20% (*w/w*) was around 33 °C which was capable to form hydrogels *in vivo* since the temperature of vitreous was around 37 °C, the concentration was preferably at 15% (*w/w*) since copolymer could satisfy the requirement of both syringeability and sol–gel transform temperature at this concentration. Therefore, the concentration of 15% (*w/w*) was selected for the following *in vivo* study.

### *In vitro* degradation of PBLA-PEG-PBLA copolymer

**Figure 3.**
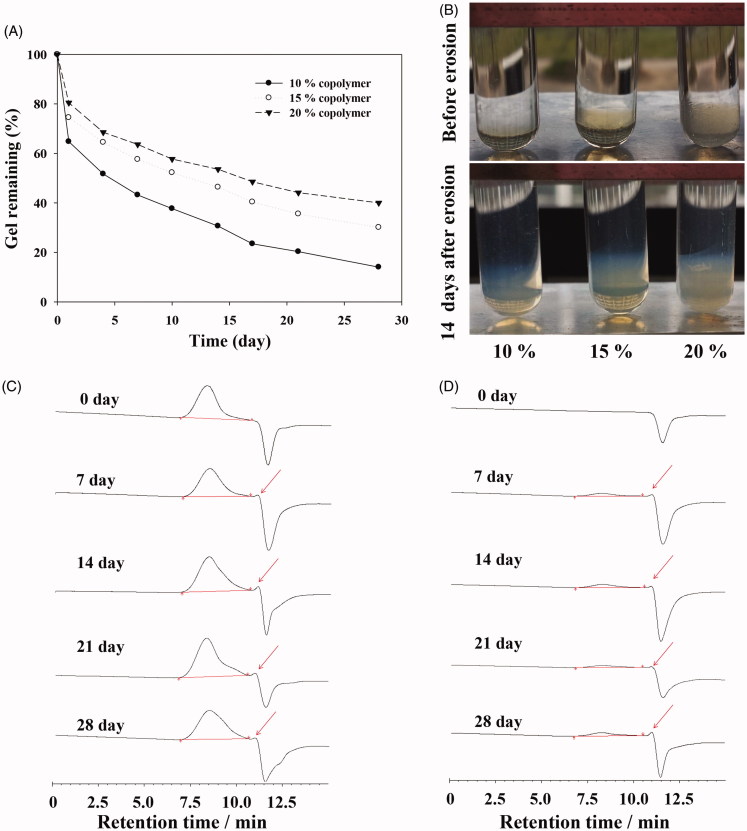
(A) Percent of the remaining hydrogel during *in vitro* degradation in PBS at 37 °C. The concentrations of polymer were 10, 15, and 20% (*w/w*), respectively. (B) Morphology of PBLA-PEG-PBLA hydrogel with different polymer concentration 10, 15, and 20% (*w/w*). (C) GPC chromatograms of the remaining gels during *in vitro* degradation. (D) GPC chromatograms of the degraded hydrogels during *in vitro* degradation.

The *in vitro* degradation of hydrogel was monitored by the changes in weight and *M_w_* in PBS (pH 7.4) during incubation at 37 °C in water bath. Degradation process would cause the loss of the weight for the hydrogel. As shown in Figure 3(a), as the PBLA-PEG-PBLA copolymer concentration was 15% (*w/w*), the hydrogel was degraded as debris on the surface and the weight was decreased quickly in the first day with about 15% of the gel degrading. From day 2, the degradation percentage of hydrogel was reached to about 2% per day for 28 days. During the degradation process, softening and increased fluidity of the hydrogel were observed after day 14 (Figure 3(b)). Over 50% of the amount of hydrogel was degraded in day 14. Meanwhile, a faster degradation was also observed for 10% hydrogel group on the first day than that of 15% hydrogel group (*p* < .05). But, from day 2, there is no significant difference in the rate of degradation between the 10% hydrogel group, 15% hydrogel group and 20% hydrogel group. It indicated that the degradation or dissolution rate of gel was not related to the concentration of copolymers except the first day which was caused by different hydrogel strengths.

The *M_w_* of the remaining copolymers determined by GPC was relatively stable during the *in vitro* degradation evaluation (Figure 3(c)). Although during the 28 days degradation, the shape of copolymer peaks and retention times shows no significantly change from the original ones, but only appears smaller peaks of degradation products at 11 min, which indicated the PBLA-PEG-PBLA can be degraded to small molecules compounds with PBS environment (pH 7.4). But the dissolution of hydrogel is not only described as all hydrophobic block of copolymers molecules hydrolysis but also include hydration and then dissolution of copolymer as shown in Figure 3(d). After the detection of degraded hydrogels by GPC, the spectrums were both shown copolymer peaks and degradation product peaks during the whole experimental process, which demonstrated PBLA-PEG-PBLA copolymer could be degraded and dissolved at same time. Those two mechanisms were both contributed to the *in situ* mass decrease and therefore we speculate PBLA-PEG-PBLA copolymer was biodegradable through dissolution of hydrogel and hydrolysis of hydrophobic block of copolymers.

### Characterization of GCV in situ hydrogels

**Figure 4.**
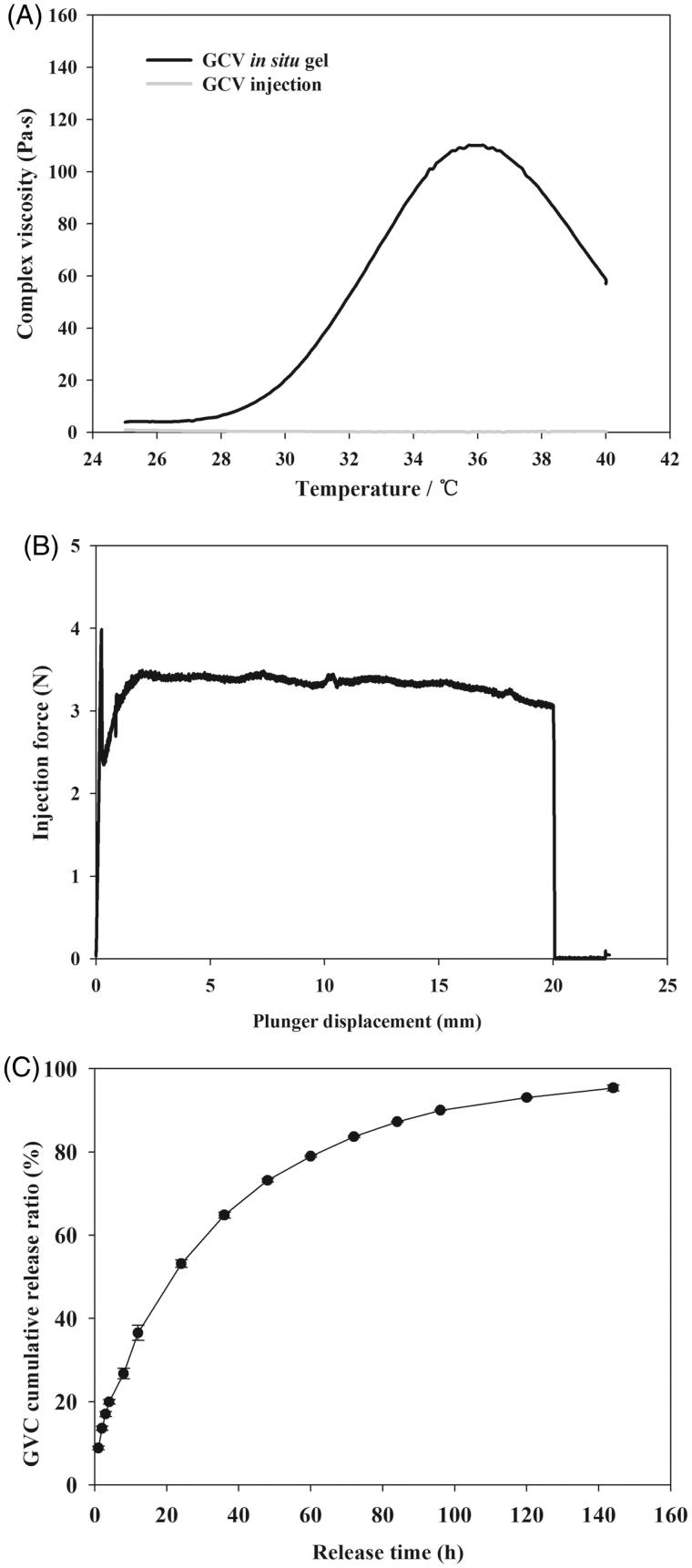
(A) Viscosity of GCV *in situ* hydrogel as a function of temperature. (B) Required force to inject GCV *in situ* hydrogel from 1 mL syringers equipped with 27 G × 12.9 mm needles into air at the crosshead speed of 1 mm·s^−1^. (C) *In vitro* GCV release profiles from GCV *in situ* hydrogel in phosphate buffer solution (PBS, pH 7.4) at 37 °C with 1% hyaluronic acid (HA, *w/w*).

GCV-loaded *in situ* hydrogels were prepared as literature reported before (Xu et al., 2014b). Briefly, PBLA-PEG-PBLA copolymer, GCV, mannitol, and sodium chloride were dissolved in phosphate buffer (pH 7.4) and passing through a microporous membrane filter (0.22 μm). The drug-loaded rate, gelation temperature, pH, and osmotic pressure of three batches of GCV hydrogels (Table S4) were characterized. The gelation temperature of GCV *in situ* hydrogels was about 30.5 °C and the highest viscosity at 36 °C which can ensure to form hydrogel at human body temperature (Figure 4(a)). The pH values were in the tolerance range of eyes which was 6.0–8.0 (National Pharmacopoeia Committee, 2015), and the *in situ* hydrogels were isotonic to minimize the irritation of the eyes. The changes of gelation temperatures were caused by adding mannitol and sodium chloride as osmo-regulator. Related researches indicated that the addition of small molecule osmo-regulator can lead to a decrease of the gelation temperature (Gilbert et al., 1987; Edsman et al., 1998). We hypothesized that the small molecule osmo-regulator would inhibit the hydrogen bond between PEG and water molecules. Inorganic slats like NaCl, NaH_2_PO_4_, Na_2_CO_3,_ and so on dissolved in water to form ions and water molecules has more affinity for the ions than the PEG shell of copolymer micelle. The number of free water molecules which can form hydrogen bond with PEG chains were decreased leading to the decreased solubility of PBLA-PEG-PBLA copolymer which caused the sol–gel transition temperature decreased.

At room temperature, the hydrogel had comparable viscosity as the aqueous solution and can be injected smoothly (Figure 4(b)). The obtained value of *F*_max_ and DGF are smaller than 5 N which satisfy the requirements of syringeability. Taken together, the thermosensitive hydrogel formulation ensured good injectability and suitable gelation temperature which formed the hydrogel depot quickly once injected *in vivo*.

### *In vitro* release

Cumulative release curve of GCV from *in situ* hydrogels were plotted against the release time in Figure 4(c). More than 80% of GCV were released in 120 h for *in situ* hydrogels. The four kinetic models (zero-order release model, first-order release model, Higuchi diffusion model, and Ritger–Papper model) were utilized to investigate the release mechanism of GCV-loaded *in situ* hydrogels. As shown in Table S5, the Ritger–Papper model was the best fit to the obtained release profile. The characteristic exponent *n* of Ritger–Papper model is 0.4679, indicating that the releasing mechanism of GCV *in situ* hydrogels was a synergistic effect of non-Fickian diffusion and gel matrix erosion. A burst release was observed in the first 12 h. This could be explained by the good solubility of GCV (4.3 mg·mL^−1^ in water, 25 °C) and that GCV in the outlayer of the hydrogels could easily diffuse out in the beginning, followed by the slow expansion of the hydrogel. Instead of embedding all GCV into the hydrophobic network of the hydrogels, a part of drugs had hydrophilic parts, which were initially released with ease. Remaining drugs were released due to the degradation of hydrogel matrix. After the burst release part, GCV continued to release and reached the plateau after 120 h. The final release rate was over 90%.

### Cytotoxicity and in vivo irritation

**Figure 5.**
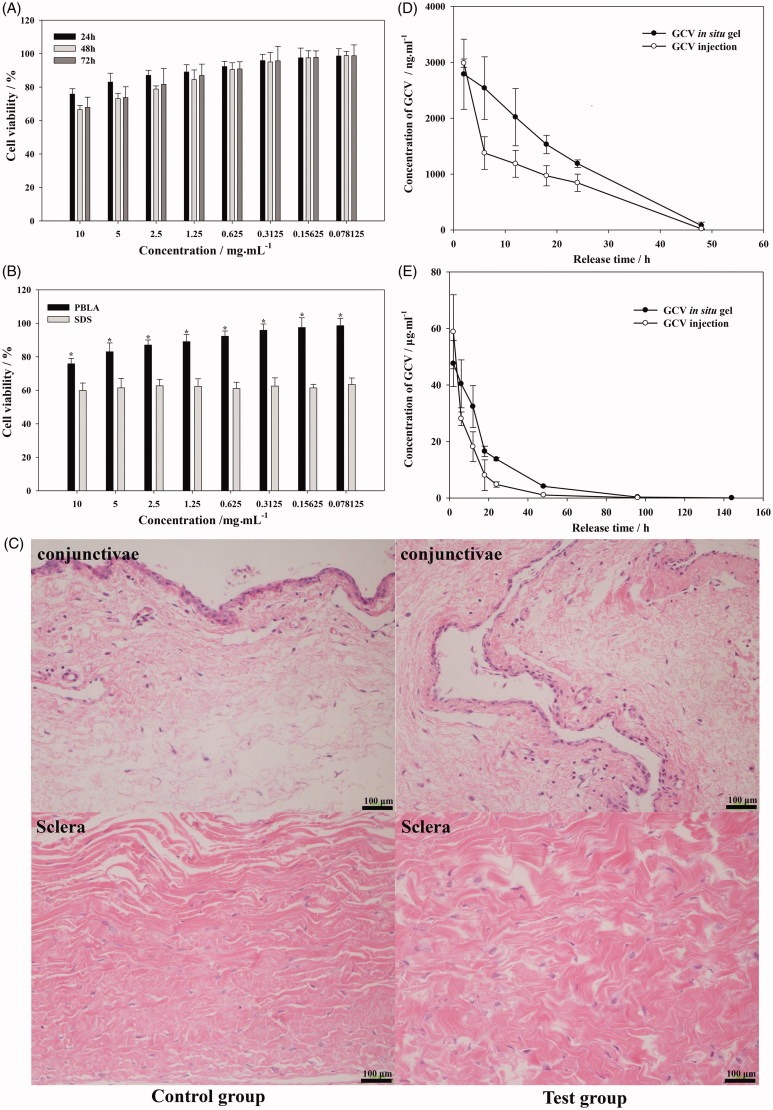
Viability of L-02 cell culture with different concentration of PBLA-PEG-PBLA (A) and compare with SDS at 24 h (B); (C) histology of conjunctivae and sclera 2 weeks after intravitreous injection. Sections were embedded in paraffin and stained with H & E; (D) aqueous humor and (E) vitreous concentration-time profile of GCV after intravitreous injection of GCV *in situ* hydrogel and GCV injection in rabbits, respectively, (*n* = 6).

In order to confirm the *in vitro* safety of materials, L-02 cell was used to research the cytotoxicity of PBLA-PEG-PBLA by MTT assay. The viability of L-02 cell was higher than 90% 24 h after culturing with PBLA-PEG-PBLA with the concentration lower than 1.25 mg·mL^−1^ (Figure 5(a)). After 48 and 72 h culture, L-02 cell also showed high cell viability (over 90%) at same concentration range, which indicated PBLA-PEG-PBLA was non-cytotoxic for liver cells when dissolved and absorbed *in situ* circulatory system. There was significant improvement on cell viability between PBLA-PEG-PBLA and positive control group (SDS) with cell viability over 70% at 24 h with PBLA-PEG-PBLA concentration was 10 mg·mL^−1^ (Figure 5(b)). According to the free concentration of PBLA-PEG-PBLA was not higher than 2.5 mg·mL^−1^ (the volume of vitreous was about 6.5 mL), which indicated PBLA-PEG-PBLA was a safe hydrogel material.

The biocompatibility of PBLA-PEG-PBLA copolymer was investigated in eye balls of rabbits. Hematoxylin-eosin tissue slice of eye balls were observed for 2 weeks after intravitreous injection (Figure 5(c)). As sclera and conjunctivae were directly in contact with the vitreous and the injection site separately, their tissue irritation was both evaluated. The sclera was mainly composed of fibroblasts with little vessels exist and was a tight structure to protect the eyeball. The conjunctiva was covered by smooth eyeball during the oculogyration. During the intravitreous injection, needle was inserted into vitreous through the sclera and therefore, both sclera and conjunctiva could contact with the hydrogels. Two weeks after injection, there was no inflammatory response (edema and inflammatory cells infiltration) observed on conjunctivae and sclera in test group which indicated PBLA-PEG-PBLA copolymers and its degradation products shown an acceptable *in vivo* biocompatibility.

### Pharmacokinetics of GCV

The concentration-time profiles of GCV in aqueous humor and vitreous after intravitreal injection of GCV *in situ* hydrogels and GCV solution at a dose of 0.2 mg·kg^−1^ were depicted in Figure 5(d,e), respectively. The GCV concentration of *in situ* hydrogels group in aqueous and vitreous was higher than that of aqueous solution group at almost all time-points (except 2 h) because of the network structure formed by copolymer hydrogels leading to prolonged retention time in the vitreous tissue and eventually enhanced the bioavailability of GCV. For both hydrogel and solution groups, the max concentration of GCV in vitreous was much higher (19.73 and 17.05 times, respectively) than that in aqueous humor, indicating that drugs were mainly absorbed to the posterior segment of eyes and then excreted through aqueous fluid circulation.

Table 1 summarized the results of the pharmacokinetics parameters. The AUC of GCV *in situ* hydrogel preparation (61.80 μg·mL^−1^·h (*p* < .01) and 1008.66 μg·mL^−1^·h (*p* < .01)) was significantly higher than that of GCV injection (41.77 μg·mL^−1^·h and 588.86 μg·mL^−1^·h) in both aqueous and vitreous. The AUC of GCV in vitreous by injecting hydrogel were significantly higher (16.32 times and 14.10 times, respectively) than that in aqueous humor, also indicating that the drugs were mainly sustained and absorbed in the vitreous cavity. *t*_1/2_ of GCV *in situ* hydrogels preparation was higher than that of GCV injection in vitreous (*p* < .05) but showed no difference in aqueous humor. The concentration of GCV for hydrogel group in aqueous humor and vitreous was still higher than the median effective concentration (ED 50, 0.23 μg·mL^−1^ to HSV-I) after 144 h of injection (Macha & Mitra, 2002; Janoria et al., 2009). By comparing with the *in vitro* release curve, the greater prolonged retention of GCV was observed in vitreous for hydrogel group, which indicate that *in situ* hydrogels has strong function of control release *in vivo* environment.

**Table 1.** Pharmacokinetics parameter of GCV in aqueous humor and vitreous.

	Aqueous humor		Vitreous	
Tissue sample	GCV *in situ* gel	GCV injection	GCV *in situ* gel	GCV injection
*C*_max_/μg·mL^−1^	2.79 ± 0.63	2.98 ± 0.07	47.58 ± 8.13	58.82 ± 13.11
*t*_1/2_/h	10.29 ± 0.51	9.48 ± 0.71	13.26 ± 0.82**	8.75 ± 0.84
AUC/μg·mL^−1^·h	61.80 ± 4.43**	41.77 ± 4.12	1008.66 ± 62.12**	588.86 ± 44.42

Significantly different vs. GCV injection, *p < .05, **p < .01 (*n* = 6).

## Conclusions

In this study, the potential to use composite PBLA-PEG-PBLA-based *in situ* thermosensitive hydrogels for the intravitreal delivery of GCV was proposed and studied. The developed PBLA-PEG-PBLA hydrogel was quickly formed at physiological conditions of 37 °C with 15% concentration (*w/w*). Rheological experiments demonstrated that the copolymer solution could transform to hydrogel at temperature range between 33 °C and 38 °C. Incorporating GCV into PBLA-PEG-PBLA solution did not affect the sol–gel transition window under physiological condition and syringeability was also satisfactory. *In vitro* cytotoxicity results showed the higher cell viability of the copolymer compared to positive control group, which provided satisfied safety for its *in vivo* applications. No significantly inflammatory response occurred in the conjunctivae and the sclera during the *in vivo* tissue irritation evaluation. *In vitro* and *in vivo* release results showed a remarkable sustained release effect. In conclusion, the ophthalmic thermosensitive *in situ* hydrogel showed good stabilities and nonirritant, which met the requirements of intravitreal use. *In vitro* release and *in vivo* pharmacological studies indicated that ophthalmic thermosensitive *in situ* hydrogel could prolong the drug release and enhance the ocular bioavailability.

## Supplementary Material

Supporting_informationIDRD_1413448.docx
