# Supplementary material for: Synthesis, physicochemical properties and ocular pharmacokinetics of thermosensitive in situ hydrogels for ganciclovir in cytomegalovirus retinitis treatment
Source: Drug Deliv. 2017 Dec 12;25(1):59–69. doi: 10.1080/10717544.2017.1413448 (PMC6058567; doi:10.1080/10717544.2017.1413448)
Supplement: Supporting_informationIDRD_1413448.docx [file IDRD_A_1413448_SM0563.docx]

Supporting information

**Synthesis, physicochemical properties and ocular pharmacokinetics of thermosensitive *in situ* hydrogels for ganciclovir in cytomegalovirus retinitis treatment**

Qiyue Wang ^1#^, Chunmeng Sun ^1#^, Bohui Xu^2^, Jiasheng Tu ^1,*^, Yan Shen^1,*^

**Materials and methods**

***Investigation of PBLA-PEG-PBLA copolymer gel solutions property***

*Preparation of PBLA-PEG-PBLA copolymer gel solutions*

Copolymer gel solutions were prepared by dissolving PBLA-PEG-PBLA copolymers in phosphate buffer (pH 7.4) with stirring at 5 %, 10 %, 15 %, 20 % and 25 % (*w/w*).

*Gelation temperature*

The gelation temperatures of copolymer solutions were determined by vial inverting method. Vials containing 3 mL of copolymer gel solutions were immersed into a thermostatic water bath with a temperature ramp at the rate of 0.5 °C·min^-1^ and an equilibrium time of 5 min. The gelation temperature of hydrogels was measured by inserting a thermometer into the immobile gel within 30 s.

*Gelation time*

The gelation time of copolymer solutions were determined by vial inverting method and magnetic stirring method. For the inverting method, vials containing 3 mL of copolymer gel solutions were immersed into a SHZ-82A thermostatic water bath (Changzhou Guohua Electric Appliance Co. Ltd, China) with a temperature at 37 °C and start timing. Then stop timing after the hydrogel was immobile and the gelation time was obtained. For the magnetic stir method, a magneton was put into vials containing 3 mL of copolymer gel solutions. Vials were immersed into a thermostatic water bath (37 °C) with a stirring rate 25 rpm and start timing. Then stop timing after the magneton was immobile and the gelation time was obtained.

*Morphology of PBLA-PEG-PBLA in situ hydrogels*

The morphology of copolymer solutions were observed by the UB200i light microscope (Nan Jing Ji Fei Technology Co., Ltd., Nan Jing, China). Copolymer solution with different concentration (10 %, 15 % and 20 %, *w/w*) was injected into PBS solution (pH 7.4) at 37 °C via 1 mL size syringes and the digital camera was used to record the process of formation of PBLA-PEG-PBLA *in situ* hydrogels. Also, the copolymer solution was dropped on the slide glass substrate and cover slide was covered and removed the air bubble. The slide glass substrates were maintained in a 37 °C environment temperature for 30 min to form the hydrogel structure and then were observed to confirm the gel structure by the light microscope. Images were acquired using JFMV300CG camera lens and JFMV controller software (Nan Jing Ji Fei Technology Co., Ltd., Nan Jing, China).

***Cytotoxicity of PBLA-PEG-PBLA***

Human normal hepatocytes (L-02) were cultivated in Roswell Park Memorial Institute 1640 Medium at 37 °C, 5 % CO_2_ condition, which all supplemented with 10 % (*v/v*) fetal bovine serum (FBS) and 1 % penicillin. A log-phase growth cells were counted, diluted and then seeded in 96-well plates at 5×10^3^ cells per well (5×10^4^ cells·mL^-1^). The plates were pre-incubated for 12 h and further incubated with 100 μL of DMEM complete medium contains different concentration of FDKP (FDKP concentration range from 0.15625 - 20 mg·mL^-1^) for 24 h, 48 h and 72 h. 10 μL of MTT solution (5 mg·mL^-1^) was added in each well and removed out after continue culturing other 4 h. 150 μL of dimethyl sulfoxide was added in each hole for dissolving MTT crystal 10 min and the optical density was determined at 490 nm in a ELx800® microplate reader (BioTek Instruments, Inc., Winooski, US).

***Tissue irritation***

Two rabbits were anesthetized with an ear vein injection of 20 % (*w/w*) urethane solution (5 mL·kg^-1^) and divided into two groups. Then, 0.1 mL of PBLA-PEG-PBLA copolymer solution (15 %, *w/w*) was injected into the vitreous cavity of rabbit using a syringe with a 27 G needle as a test group. The normal saline was also injected as control group. The animals were euthanized at the 14^th^ day (2 weeks) after injection. Eye balls were removed and then stored in 10 % formalin solution (*v/v*) at 4 °C for fixation and then transferred to 50 % ethanol (*v/v*). Sections (5 μm) were prepared, stained with hematoxylin and eosin (H&E), and examined by light microscopy. Images were acquired using JFMV300CG camera lens and JFMV controller software (Nan Jing Ji Fei Technology Co., Ltd., Nan Jing, China).

Table S1 Formulation of PBLA-PEG-PBLA copolymer *in suit* hydrogel solution

| Materials | Concentrate (%) | Function |
| --- | --- | --- |
| copolymers | 15 | Temperature-sensitive gel material |
| ganciclovir | 0.2 | Active Pharmaceutical Ingredient |
| Gelatin | 0.25 | freeze-drying protective agent |
| Mannitol | 2 | freeze-drying protective agent |
| Sodium chloride | 0.4 | Osmotic regulator |
| phosphate buffer solution (0.05 mol·L^-1^) |  | pH regulator and solvent |

**Results and discussion**

***^1^H NMR and GPC spectra of PBLA-PEG-PBLA copolymer***

Table S2 Molecular weight and PDI of PBLA-PEG-PBLA

| Test | *M*_n_ | *M*_w_ | PDI |
| --- | --- | --- | --- |
| 1 | 4334 | 6378 | 1.472 |
| 2 | 4259 | 6281 | 1.475 |
| 3 | 4198 | 6073 | 1.447 |
| Mean±SD | 4263 ± 68 | 6244 ± 156 | 1.464 ± 0.015 |

***Rheology, syringeability and morphological observation***

Table S3 Results of sol-gel phase transition temperature and gelation time of copolymer solution (Mean±SD, n=3)

| Concentration (*w/w*) | | 10 % | 15 % | 20 % |
| --- | --- | --- | --- | --- |
| Gelation temperature(°C) | Reversing method | 34.0 ± 0.3 | 33.0 ± 0.3 | 32.5 ± 0.2 |
|  | Rheology method | 33.01 ± 0.21 | 32.37 ± 0.16 | 31.08 ± 0.27 |
| Gelation time(s) | Reversing method | 30.17±4.58 | 15.50±2.17 | 6.67±1.51 |
|  | Magnetic stirring method | >100 | 31.67±13.19 | 17.17±7.55 |


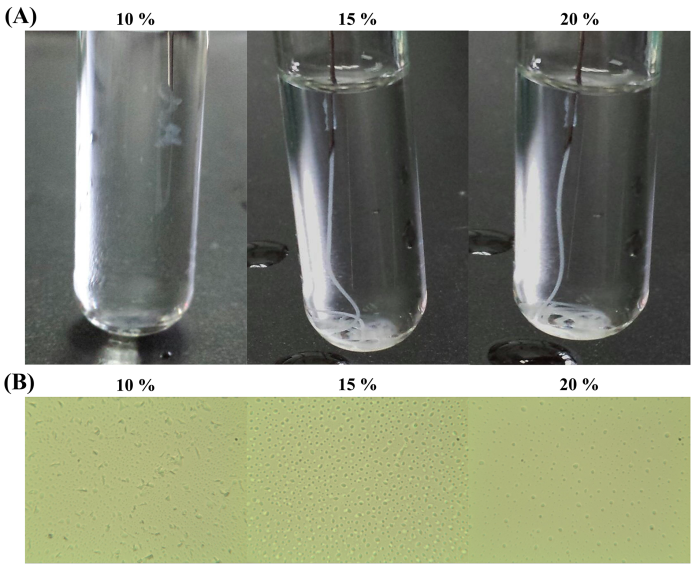


Figure S1. (A) Forming image of PBLA-PEG-PBLA hydrogel with concentration of 10 %, 15 % and 20 % during inject; (B) Microscopic structure of PBLA-PEG-PBLA hydrogel with concentration of 10 %, 15 % and 20 % after moisture evaporation.

***Characterization of GCV in situ hydrogels***

Table S4 Results of basic physicochemical properties of ganciclovir *in situ* hydrogel.

| Batches | 1 | 2 | 3 | Mean ± SD |
| --- | --- | --- | --- | --- |
| Drug loaded rate (%) | 1.11 | 1.12 | 1.09 | 1.11 ± 0.02 |
| Gelation temperature(°C) | 30.6 | 30.4 | 30.6 | 30.5 ± 0.1 |
| pH | 6.17 | 6.13 | 6.11 | 6.14 ± 0.03 |
| osmotic pressure(mOsm·kg^-1^) | 305 | 312 | 307 | 308 ± 4 |

***In vitro release***

Table S5 Release study on fitting of kinetic model

| release model | Model | formulas | r | n |
| --- | --- | --- | --- | --- |
| Zero-order release model | Q=kt | y=0.5246x+24.63 | 0.9342 | / |
| first order release model | Q=1-e^-kt^ | y=0.0119x+3.0986 | 0.8274 | / |
| Higuchi equation model | Q=kt^1/2^ | y=7.5593x+5.6528 | 0.9912 | / |
| Ritger-Papper equation model | Q=kt^n^ | y=0.4679x+2.2727 | 0.9962 | 0.4679 |
